# Differential Roles of Astrocytic CSF1 in Alzheimer's Disease and Cerebral Amyloid Angiopathy: Insights from Transcriptomic Analysis

**DOI:** 10.14336/AD.2024.10530

**Published:** 2025-11-05

**Authors:** Chunyuan Li, Yashuang Chen, Shiqi Luo, Yan Yang, Xinnan Liu, Sijie Li, Wei Ge, Cong Han

**Affiliations:** ^1^The State Key Laboratory for Complex, Severe, and Rare Diseases, Department of Immunology, Institute of Basic Medical Sciences Chinese Academy of Medical Sciences, School of Basic Medicine Peking Union Medical College, Beijing, China. ^2^Department of Neurology, Xuanwu Hospital, Capital Medical University, Beijing, China; Department of Emergency, Xuanwu Hospital, Capital Medical University, Beijing, China; Beijing Institute of Brain Disorders, Capital Medical University, Beijing, China. ^3^Department of Neurosurgery, First Medical Center of Chinese PLA General Hospital, Beijing, China.

**Keywords:** Transcriptomics, Alzheimer’s disease;, Cerebral amyloid angiopathy, Astrocyte, CSF1

## Abstract

Alzheimer's disease (AD) and cerebral amyloid angiopathy (CAA) are neurodegenerative disorders characterized by the pathological deposition of amyloid-beta (Aβ) in the brain. Although both conditions share common pathogenic pathways, they exhibit distinct cellular manifestations and disease progression. This study focused on the differential expression and role of astrocytic colony-stimulating factor 1 (CSF1) in these diseases. Through transcriptomic analysis of 248 brain tissue samples from the hippocampal-entorhinal system of 50 individuals, we identified a significant increase in CSF1 expression in the CA4 subfield of AD patients, contrasting with a marked decrease in CAA. Functional investigations revealed that astrocytes with elevated CSF1 levels displayed neurotoxicity associated with AD-like pathology, while reduced CSF1 expression in astrocytes was linked to vascular damage characteristic of CAA. These findings suggest that CSF1 plays divergent roles in AD and CAA, contributing to their distinct pathological profiles. Our study highlights the potential of targeting astrocytic CSF1 expression as both a differential diagnostic marker and a therapeutic approach in managing these overlapping yet distinct neurological conditions.

## Introduction

Amyloid-β (Aβ) is a product of the proteolytic cleavage of amyloid precursor protein (APP) and plays a role in signal transduction within the brain. Dysregulation of Aβ levels is a recognized key factor in various neurodegenerative disorders, such as Alzheimer's disease (AD) and cerebral amyloid angiopathy (CAA).

Neuropathological and tracer studies indicate that AD and CAA share overlapping pathogenic pathways involving Aβ production, its circulation in interstitial fluid and perivascular drainage pathways, and its clearance from the brain. A reduction in perivascular clearance of Aβ is a common pathogenic mechanism in both conditions [1]. Clinically, advanced CAA in AD is associated with greater cognitive impairment and faster cognitive decline, suggesting a shared contribution to clinical dysfunction [2,3]. Given these overlaps in cognitive impairment [3], nearly every clinical trial targeting sporadic CAA or AD can be considered a therapeutic trial for both conditions [4].

Although the co-occurrence of CAA and AD is more common than other vascular and neurodegenerative pathologies, they do not entirely synchronize [5]. Studies suggest that AD and CAA differ in the mechanisms underlying brain injury and disease presentation. AD-related injury primarily involves Aβ-induced synaptic and neuronal loss and the development of hyperphosphorylated tau neurofibrillary lesions [6], while CAA-related injury arises from vascular dysfunction, including large intracerebral hemorrhages, cerebral microbleeds, and cortical sulcal hemorrhages [7]. Neuroimaging findings also show that CAA-associated vessel ruptures can occur independently of AD [8]. Mutations in different regions of the APP gene have been linked to early-onset AD phenotypes or the clinical-pathological phenotype of dominant CAA [9,10].

Astrocytes play essential roles in both AD and CAA, contributing to brain functions such as synapse formation, neurotransmitter regulation, trophic factor production, and neuronal survival [11,12]. In AD, Aβ-stimulated astrocytes and microglia release neuroinflammatory factors (e.g., IL-1β, TNF-α), exacerbating brain pathology. Astrocytes encase the blood-brain barrier (BBB) endothelial cells and form a lamellar network near the endothelium’s outer surface, creating the perivascular space, which is implicated in hemorrhagic events in CAA [11]. Additionally, the outermost layer of astrocytes surrounding vessels, known as the glia limitans, contains aquaporin 4 (AQP4), a channel critical for cerebrospinal fluid movement into the brain parenchyma [13].

Colony-stimulating factor 1 (CSF1) orchestrates the proliferation and differentiation of myelomonocytic cells and plays a crucial role in initiating and maintaining the macrophage immune response [14]. Astrocytes, oligodendrocytes, and microglia can express CSF1, which binds to CSF1R to participate in pathophysiological processes [15]. Elevated CSF1 and CSF1R levels have been observed in brain tissues of individuals with AD pathology [16,17]. CSF1 promotes the secretion of proinflammatory cytokines, inducing neuroinflammation [18]. However, the signaling mechanism by which CSF1 contributes to the differing brain damage and clinical presentations of AD and CAA remains unclear.

In this study, we performed transcriptome analysis on brain tissues from the hippocampal-entorhinal system subfields, based on samples from the National Human Brain Bank for Development and Function. We identified a distinct expression pattern of astrocytic CSF1 in the CA4 region between AD and CAA. Our findings provide a novel perspective for the early diagnosis of these two typical vascular and neurodegenerative diseases with overlapping yet distinct effects.

## MATERIALS AND METHODS

### Human brain tissue collection

Human brain tissue samples were obtained from the National Human Brain Bank for Development and Function, Chinese Academy of Medical Sciences, and Peking Union Medical College, Beijing, China. The brain tissues were collected following the international standard human brain banking procedure. Before collection, it is necessary to confirm the time and cause of death of the donor, obtain the consent signature of the family members, record the manifestations before death and evaluate the risk factors. If the samples cannot be collected in time, the body will be cryopreserved, and the post-mortem interval will be recorded. The procedure for brain tissues collection is as follows: (1) confirm the brain collection time; (2) determine whether there is craniocerebral trauma; (3) expose the skull coronally; (4) open the skull; (5) disconnect the brain from the skull; (6) remove the brain completely; (7) temporarily store in ice isotonic saline and take pictures; (8) decide on the processing process.

After the whole brain was collected, the weight, volume and length were measured, and it was observed whether it was symmetrical and whether there were visible surface injuries. If so, the damaged half needs to be fixed and stained, and pathological diagnosis is performed; the contralateral brain is frozen and fixed. In addition, the samples were preserved by rapid low-temperature freezing of brain hemisphere slices and formalin fixation of the brain hemispheres.

### Ethical statement on human brain tissue samples

Ethical review of brain tissue samples used in this study has been approved by the Ethics Committee of the Institute of Basic Medical Sciences, Chinese Academy of Medical Sciences (Project No. 009-2014). The samples are under the supervision and management of the Institute of Basic Medical Sciences and the Neuroscience Center. All brain tissues are obtained from voluntary donations made during the donor’s lifetime. Once all family members of the donor have reached a consensus on the donation, the donor completes the donation registration form in accordance with the prescribed procedures, and the process is notarized. Throughout the process, the donor provides informed consent, and the donation registration form holds the same legal effect as the informed consent form.

Regarding data privacy, the brain bank established a unified database with tiered access permissions for different personnel. The brain bank is responsible for maintaining the confidentiality of donors’ information. Once collected by brain bank, donor tissues are immediately coded to prevent information leakage. When samples are provided to researchers, only the code and pathological information are shared. The researchers then further re-code the samples to ensure that no additional information is disclosed.

### Transcriptome sequencing

The sequencing libraries were generated using the VAHTS Total RNA-Seq Library Prep Kit for Illumina (NR603-02, Vazyme) according to the manufacturer’s instructions. The samples were paired-end sequenced on an Illumina HiSeq 2000 sequencer. The process of sequencing was controlled by Illumina Data collection software. The library construction and sequencing were performed at Shanghai Biotechnology Corporation.

Our Bulk RNAseq data was deposited to the Gene Expression Omnibus (GEO) database (www.ncbi.nlm.nih.gov/geo/query/acc.cgi?acc=GSE278723) with the dataset identifier GSE278723.

### Differentially expressed gene (DEGs) analysis

The differentially expressed mRNAs in each of the five subfields of the hippocampal-entorhinal system were evaluated using the DESeq2 R package [19], and the original count values were as input data. DEGs were identified based on the criteria of |log2(Foldchange)| > 0.58 and the p value < 0.05.

### Single-cell transcriptome analysis and deconvolution

The raw single-cell data for the five subfields of the human hippocampal-entorhinal system were obtained from the GEO database under the accession number GSE186538 (www.ncbi.nlm.nih.gov/geo/query/acc.cgi?acc=GSE186538). The single-cell analysis was conducted based on methodologies described in the previous study [20]. In brief, 8,000 cells were randomly selected from the total cell population. The data were then processed, including cleaning, normalization, scaling and performing principal component analysis (PCA), using the Seurat R package [21]. Cell types were clustered using known cell-type markers, containing oligodendrocyte, oligodendrocyte precursor cell (OPC), neuron, astrocyte, endothelial cell, and microglia. The clustered data were then deconvoluted using the CIBERSORTx [22], beginning with the construction of the signature matrix. Subsequently, the signature matrix and RNA-Seq normalized counts per million (CPM) data for each subfield were used to deconvolute. S-mode batch correction and 100 permutations for significance analysis were applied, with quantile normalization disabled. The cell proportions obtained from each subfield were grouped into "NC", "AD","CAA", and "AD+CAA" categories. The variations in the proportions of different cell types in each group were visualized using violin plots.

### Weighted Gene Co-Expression Network Analysis (WGCNA)

WGCNA was performed on the dataset from the hippocampal CA4 subfield of 34 individuals using the WGCNA R package [23]. A signed network was constructed using WGCNA, with soft-thresholding power set to 14. The robustness of modules was assessed using the modulePreservation() function. A Zsummary score greater than 10 indicates a strongly preserved module; a Zsummary score >2, but <10 denotes moderate preservation. While a score less than 2 indicates a non-preserved module. The results were visualized using bubble plots created with the ggplot2 R package. Modules eigengenes were calculated using the moduleEigengenes () function. The correlation coefficient between module eigengenes expression and AD or CAA pathological levels were assessed using Pearson correlation analysis.

### Cell-type enrichment analysis

Cell-type-specific markers were sourced from the PanglaoDB database [24]. Fisher’s exact test was used to determine whether the genes in each WGCNA module were enriched in specific cell types, including astrocyte, endothelial cell, microglia, neuron, and oligodendrocyte. The enrichment results were visualized using the circlize R package [25], with colors representing the *p*-value for each module.

## Bioinformatics analysis

Gene Ontology (GO) and Kyoto Encyclopedia of Genes and Genomes (KEGG) enrichment analyses were performed using the clusterProfiler R package [26], and p value < 0.05 was considered statistically significant. Protein-protein interaction (PPI) network analysis for DEGs was conducted using STRING (https://string-db.org/), and the results were visualized with Cytoscape. Principal component analysis (PCA) was conducted using the prcomp() function in R. PCA plot visualization was performed using the ggord R package (Marcus W Beck, & Vladimir Mikryukov. (2022). fawda123/ggord: v1.1.7 (v1.1.7). Zenodo. https://doi.org/10.5281/zenodo.6382531).

### Primary Astrocyte and Hippocampal neuron Culture

All animal experiments were conducted in accordance with a protocol approved by the Ethics Committee of the Institute of Basic Medical Sciences, China. Cortical astrocytes and hippocampal neurons were isolated from postnatal day 1 rats. Briefly, the rat cortices hippocampi tissues were enzymatically digested with 0.25% trypsin at 37 °C for 15 minutes. The supernatant containing the dissociated cells was passed through a 70-μm nylon mesh cell strainer into a 50-mL conical tube and centrifuged at 200 × g for 5 minutes to pellet the cells. After removing the supernatant, the cells were resuspended in Dulbecco’s Modified Eagle’s Medium (DMEM) containing 20% fetal bovine serum (FBS) and 1% penicillin-streptomycin, and then plated in 10-cm dishes. The hippocampal neurons were obtained and then seeded following by centrifugation. After 2-3 weeks of *in vitro* culture, hippocampal neurons can be used for co-culture with astrocytes.

### Cell lines

The cell lines used in this study, including the mouse astrocyte cell line C8-D1A and endothelial cell line bEnd.3, were purchased from American Type Culture Collection (ATCC). The C8-D1A cells were grown in DMEM supplemented with 10% FBS, while the bEnd.3 cells were cultivated in Roswell Park Memorial Institute-1640 medium with 10% FBS. All cells were maintained in a 5% CO_2_ atmosphere at the appropriate temperature.

### Immunostaining

Immunohistochemistry of human postmortem brain tissues was performed on 10-μm thick paraffin sections fixed with 4% paraformaldehyde. The sections were blocked with 2% bovine serum albumin (BSA) in PBS for 1 hour at room temperature before applying the primary antibody overnight at 4 °C. The human brain sections were stained with anti-GFAP (abcam, ab4648) for astrocytes. Sections were washed three times for 5 minutes each at room temperature and then incubated with DAPI (Solarbio, C0065) for 10 minutes. The sections were subsequently incubated with the secondary antibodies (Thermo Fisher Scientific, A32723) for 1 hour at room temperature after incubation with the primary antibodies. Finally, all sections were mounted on glass slides with fluorescent mounting medium (ZSGB-BIO, ZLI-9556).

The details of the immunostaining procedure of cells were as follows: cells were fixed with paraformaldehyde, then treated with 0.3 % Triton X-100 for 5 min and blocked with 5 % BSA for 30 min at room temperature. Next, the cells were incubated with anti- target protein antibody [Ki67: abcam, ab15580; GFAP: abcam, ab4648; MAP2: Cell Signaling Technology (CST), #4542; PSD-95: Synaptic Systems, 124011] or anti-IgG (Rabbit IgG, monoclonal [EPR25A] - Isotype Control: abcam, ab172730; Mouse IgG - Isotype Control: abcam, ab37355) at 4°C for overnight, followed by fluorescent secondary antibody incubation. After nuclear staining with DAPI and mounting, image acquisition was performed with a confocal microscope.

### Quantitative real time polymerase chain reaction (qRT-PCR)

Reverse transcription was performed using the PrimeScript RT Master Mix Kit (Takara, RR036A) with 500 ng of RNA per reaction. PCR was then conducted on a Bio-Rad CFX96 system with 20 ng cDNA per sample, in triplicate, using TB Green Premix Ex Taq II (Takara, RR820A). Following the PCR, melting curves of the amplified products were generated. β-actin was used as the reference gene. Relative gene expression was quantified using the 2^-ΔΔCt^ method.

### Lentivirus production and transfection

Lentivirus particles were produced in accordance with the following procedures. In brief, DNA plasmids (the detailed ratio is target DNA: Gag: Rev: VSVG = 10 : 5 : 2 : 3) were transfected into 293T cells with a confluency of 80-90% using lipofectamine 2000 reagent (Invitrogen, 11668019). Virus particles were enriched from the supernatant collected at 48 and 72 hours using PEG8000 (Sigma, P5413). The diluted lentivirus was used to infect cells for 48 hours, and the cells successfully transfected with plasmid were screened using resistance tag carried on the target gene DNA. An empty plasmid served as the negative control.

### siRNA knockdown

The non-targeting siRNA and CSF1-targeting siRNA were used in this study. And Lipofectamine™ RNAiMAX (Invitrogen, 13778150) was used for siRNA transfection in accordance with manufacturer’s instructions. Taking a 6-well plate as an example, 3×10^5^ cells were seeded in each well, and transfection was performed when the cell confluence was 70%. 9μL of Lipofectamine^®^ RNAiMAX Reagent was diluted in 150μL Opti-MEM^®^ Medium (Gibco, 31985062), and 1.5μL of 20μM siRNA was diluted in 150μL Opti-MEM^®^ Medium. Add diluted siRNA to diluted Lipofectamine^®^ RNAiMAX Reagent and incubate at room temperature for 5 minutes. The siRNA-lipid complex was added to 2mL culture medium and incubated at 37°C for 48 hours. Then, the transfected cells were analyzed.

**Figure 1.**
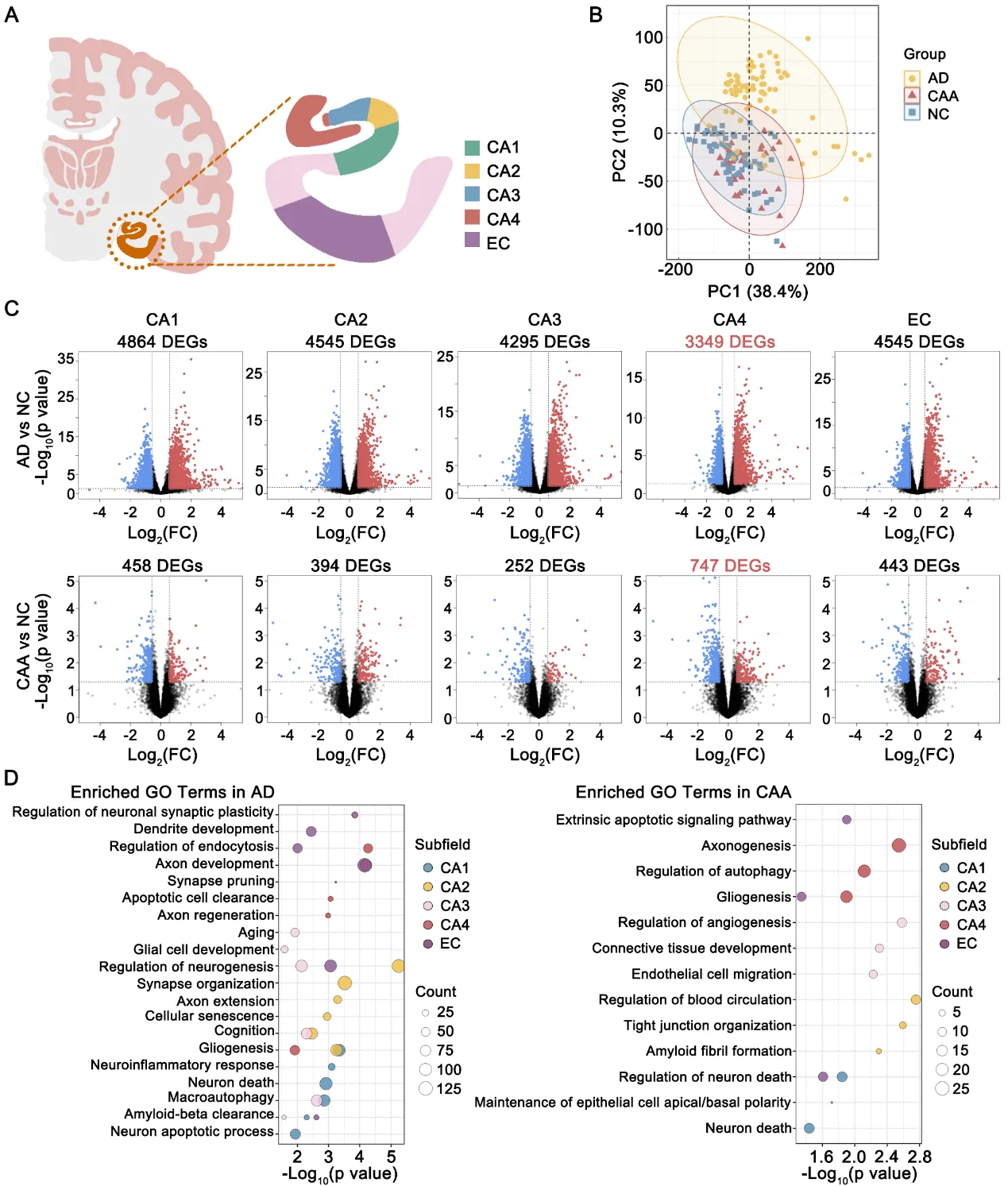
**Transcriptomic profiling of hippocampal-entorhinal system subfields from individuals with AD or CAA pathology compared with NC**. (**A**) Schematic diagram of the human hippocampal-entorhinal system anatomy. (**B**) PCA plot of RNA-Seq data from NC, AD and CAA groups. NC, n = 13; AD, n = 15; CAA, n = 6. Each color represents a group, and each dot represents a sample. (**C**) Volcano plots of DEGs for each subfield from individuals of AD and CAA groups; blue dots indicate downregulated genes and red dots indicate upregulated genes. (**D**) GO functional enrichment analyses of DEGs in hippocampal-entorhinal system subfields from AD (left) and CAA (right) groups.

### Western blot

The cells to be tested were lysed on ice, sonicated and centrifuged to measure the protein concentration. Proteins with a molecular weight of 30-80 kDa were separated using 10% SDS-PAGE gel, and proteins with a molecular weight > 100 kDa were separated using 6% SDS-PAGE gel. The polyvinylidene fluoride (PVDF, Millipore, IPFL00010) membrane carrying proteins was blocked with 5% milk and incubated with the primary antibody (CSF1: abcam, ab233387; AQP4: abcam, ab9512; ZO-1: CST, #8913; Occludin: Proteintech, 13409-1-AP; VE-Cadherin: CST, #2500; CD31: abcam, ab28364) at 4 °C. After incubation with the secondary antibody [Anti-mouse IgG (H+L), Biotinylated Antibody: CST, #14709; Anti-rabbit IgG (H+L), Biotinylated Antibody: CST, #14708] at room temperature for 1h, the protein signal was detected using ECL WB substrate (Millipore, WBKLS0500).

### Transwell migration assay

Cell migration ability was assessed using the Transwell assay. The transwell chambers (Corning, 3422) were placed in a 24-well plate, 200μL of cell suspension (2 × 10^5^ cells) resuspended in serum-free medium was added to the upper chamber, and 500μL of medium containing 20% FBS was added to the lower chamber. It is necessary to avoid air bubbles between the lower chamber’s medium and the chamber. After 72 hours, the chamber was removed and air-dried. The cells were stained with 0.1% crystal violet (Merck, C0775) at room temperature for 1 hour, followed by destaining with 33% acetic acid. Finally, the cells that migrated across membrane were captured and 5 fields of view were taken to count.

### Statistical Analysis

Statistical analyses and data visualization were performed using R v4.1.0 and GraphPad Prism 9.0. Data were presented as mean ± standard error of mean (SEM). The normality of data to be analyzed was first tested using Shapiro-Wilk test in GraphPad Prism 9.0. If the data were normally distributed, unpaired two-tailed Student’s t-test was used to compare two experimental groups; and one-way analysis of variance (ANOVA) was used to compare multiple groups. If not, nonparametric statistics were used. The statistical analyses for experimental data in this study were provided in Supplementary Table 2. P < 0.05 was considered statistically significant.

## RESULTS

### CA4 is an important hippocampal subfield affected by AD and CAA pathology

RNA sequencing (RNA-Seq) was conducted to examine gene expression in postmortem frozen human brain tissues from five subfields of the hippocampal-entorhinal system [cornu ammonis (CA) 1, CA2, CA3, CA4, and the entorhinal cortex (EC)] (Fig. 1A). Thirty-four individuals were divided into three groups: Normal Control (NC; n=13), AD (n=15), and CAA (n=6), with no significant difference in postmortem interval (PMI) between groups (Supplementary Fig. 1A). Detailed donor information is provided in Supplementary Table 1.

Principal component analysis (PCA) of the RNA-seq data was performed, showing no marked separation among CA1, CA2, CA3, CA4, and EC subfields (Supplementary Fig. 1B). However, clear separation was observed among the NC, AD, and CAA groups, particularly between AD and CAA (Fig. 1B), suggesting distinct pathological mechanisms.

To identify potential regulatory genes for each pathology, we performed differential expression analysis on genes in the five subfields of AD and CAA individuals using DESeq2. Compared with NC, we identified 4,864 differentially expressed genes (DEGs) in CA1, 4,545 in CA2, 4,295 in CA3, 3,349 in CA4, and 4,545 in EC in AD (Fig. 1C). For CAA, 458 DEGs were identified in CA1, 394 in CA2, 252 in CA3, 747 in CA4, and 443 in EC (Fig. 1C). Notably, the CA4 subfield in AD showed the fewest DEGs, while CA4 in CAA had the most (Fig. 1C). Additionally, CA4 in AD had fewer DEGs unique to this subfield, while CA4 in CAA had the most unique DEGs (Supplementary Fig. 1C). These comparisons suggest that CA4 may play a critical role in the pathological progression of AD and CAA.

To explore pathway changes associated with AD and CAA in each subfield, we performed Kyoto Encyclopedia of Genes and Genomes (KEGG) analysis on the DEGs. For AD, the Alzheimer’s disease and synapse-related signatures linked to neural communication were significantly enriched (Supplementary Fig. 1D), indicating widespread neurodegenerative molecular changes across all five subfields. In CAA, vascular endothelial growth factor (VEGF) and other pathways related to vascular function were significantly enriched (Supplementary Fig. 1D), indicating that functional changes associated with vascular endothelial damage were prominent in the hippocampal-entorhinal system.

To gain further insight into the biological functions related to AD and CAA, Gene Ontology (GO) analysis was conducted for DEGs in each subfield. For AD, terms related to synaptic function, neuron apoptosis, and glial cell function were significantly enriched (Fig. 1D). In CAA, GO analysis revealed multiple enriched terms, including amyloid fibril formation, vascular and endothelial cell function, and gliogenesis (Fig. 1D), suggesting an important role of glial cells in the pathophysiology of both AD and CAA.

**Figure 2.**
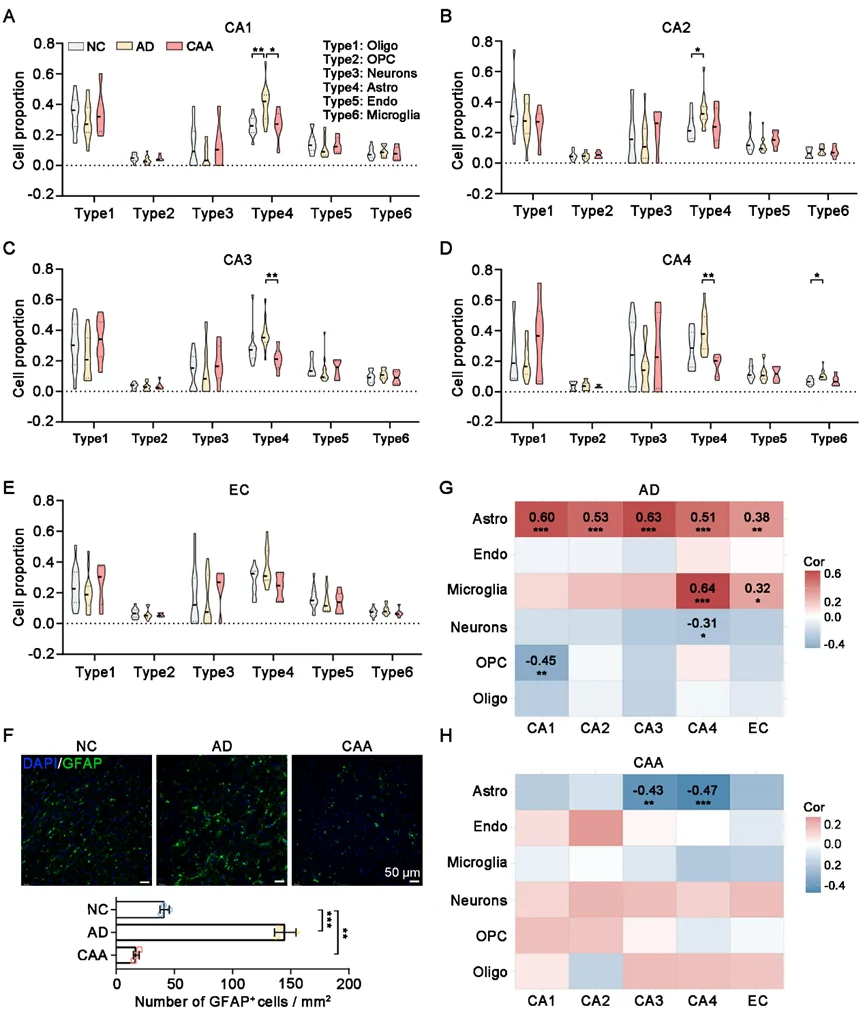
**Cell-type-specific transcriptomic changes in hippocampal-entorhinal system subfields**. (**A-E**) Individual cell-type population proportions for AD and CAA pathology in CA1 (A), CA2 (B), CA3 (C), CA4 (D), and the EC (E). Oligo = Oligodendrocyte, OPC = Oligodendrocyte precursor cell, Astro = Astrocyte, Endo = Endothelial cells. (**F**) Representative fluorescence micrographs and quantification of astrocytes in CA4 subfield of individuals with no (NC, n = 3), AD (n = 3) and CAA (n = 3) pathology. (**G-H**) Correlation heatmap of estimated cell-type proportions in bulk RNA-Seq data plotted against AD- (G) and CAA-pathology (H) score. The colors represent the coefficient of correlation. The coefficient of correlation and p values were calculated using Spearman’s correlation. *p < 0.05, **p < 0.01, ***p < 0.001, as determined by one-way ANOVA for comparing multiple groups.

### Astrocytes in CA4 are positively correlated with AD neuropathology and negatively correlated with CAA pathology

To investigate the involvement of glial cells in the pathological processes of AD and CAA, we performed deconvolution analysis on our RNA-Seq data to quantify the proportions of central nervous system (CNS) cell types using the GEO dataset (GSE186538), which includes single-cell information across the five subfields of the human hippocampal-entorhinal system (Supplementary Fig. 2). In the CA1 subfield, the estimated proportions of oligodendrocytes, oligodendrocyte precursor cells (OPCs), neurons, endothelial cells, and microglia showed no significant changes in the AD and CAA groups compared to NC. However, the estimated proportion of astrocytes was significantly higher in AD than in NC and CAA (Fig. 2A).

In the CA2 and CA3 subfields, the astrocyte proportion was also significantly higher in AD than in NC and CAA (Fig. 2B and C). In CA4, the proportion of astrocytes increased in AD and significantly decreased in CAA compared with NC (Fig. 2D), suggesting opposite alterations in astrocyte levels in CA4 for AD and CAA pathologies. In the entorhinal cortex (EC), no significant proportional changes were observed among the six cell types across the groups (Fig. 2E).

Immunofluorescence (IF) staining of astrocytes in the human hippocampal CA4 subfield validated our findings (Fig. 2F). Correlation analysis further showed that the relative abundance of astrocytes was significantly positively correlated with AD pathology scores (Fig. 2G), whereas it was negatively correlated with CAA pathology (Fig. 2H). These results suggest that the opposite changes in astrocyte proportions in the CA4 subfield may play distinct roles in promoting AD or CAA pathology.

### WGCNA combined with PPI identify key DEGs that changes in astrocytes

Considering the opposite trends in astrocyte numbers in the CA4 subfield of AD and CAA, we conducted Weighted Gene Co-expression Network Analysis (WGCNA) to identify candidate gene clusters in astrocytes. This analysis yielded 40 gene modules from 12,779 genes across 34 individuals (Supplementary Fig. 3A). To test the robustness of the WGCNA-generated network, we calculated module preservation statistics, confirming that all 40 modules were preserved (Zsummary > 2) (Supplementary Fig. 3B).

**Figure 3.**
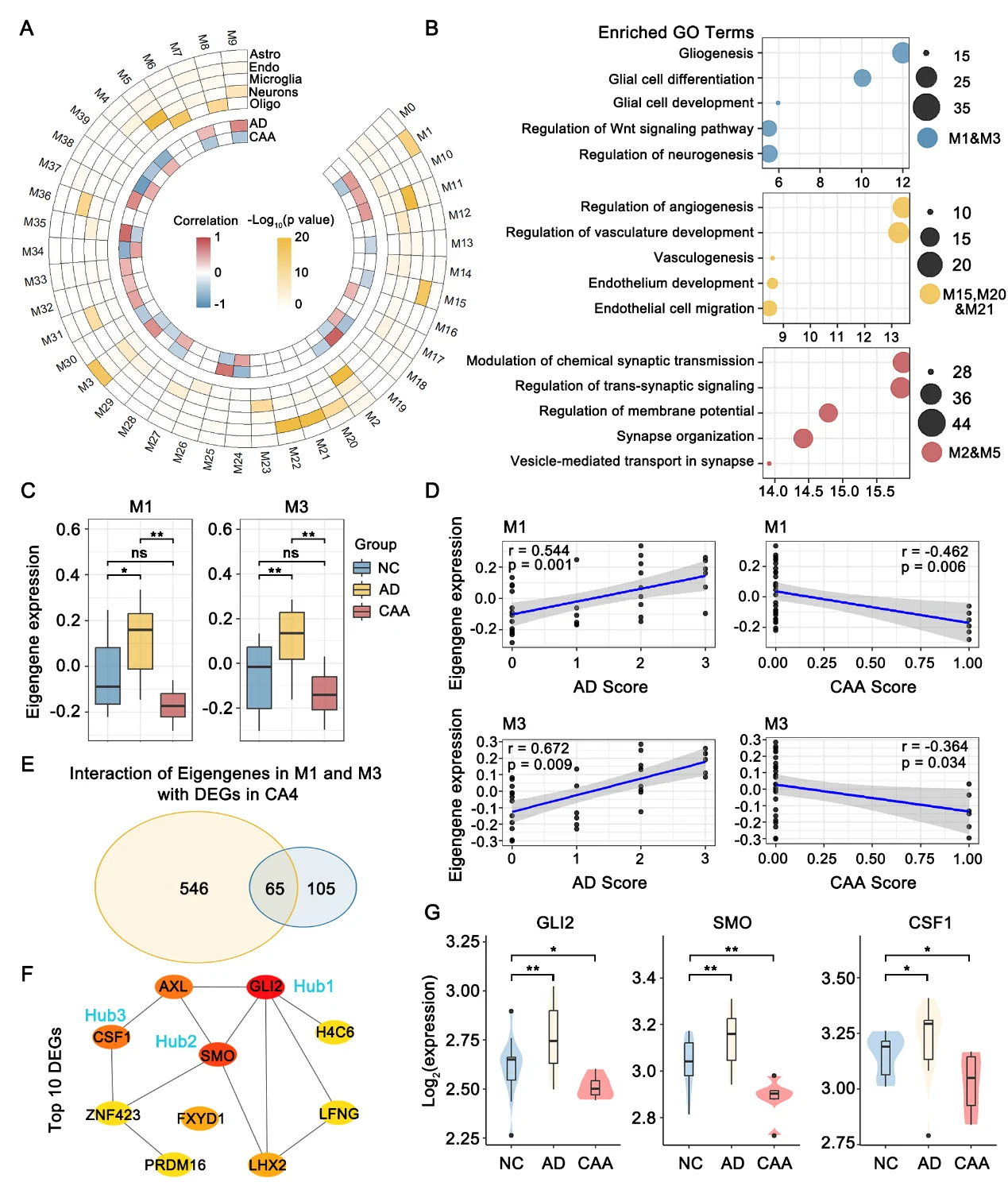
**Consensus gene co-expression network analysis across NC, AD and CAA groups and screening of astrocyte-related DEGs**. (**A**) Gene enrichment degree of cell-type-specific markers in each module. Correlation analysis between gene expression in each module and AD or CAA neuropathology. Yellow indicates adjust p value from Fisher’s exact test. Blue and red indicates correlation coefficients. (**B**) GO enrichment analysis of module genes significantly enriched in astrocytes (M1 and M3), endothelial cells (M15, M20 and M21), and neurons (M2 and M5). Colors represent modules enriched in the three cell types. (**C**) Comparison of eigengenes expression of M1 and M3 (enriched in astrocytes) in individuals with no-pathology (NC), AD pathology, and CAA pathology. (**D**) Spearman correlation analysis between the expression of eigengenes in module M1 and M3 and the pathological levels of both AD and CAA. (**E**) Venn diagram of eigengenes in module M1 and M3 and DEGs in CA4 subfield. (**F**) Visualization of the top 10 DEGs significantly associated with astrocyte functions in CA4 subfield. (**G**) Comparison of the expressions of the top 3 DEGs GLI2, SMO, and CSF1 in NC, AD, and CAA. *p < 0.05, **p < 0.01, ns = not significant, as determined by one-way ANOVA for comparing multiple groups.

To examine changes in cell-type composition in CA4 with the progression of AD or CAA, we quantified the relationships between genes in each module and AD or CAA by plotting modules enriched with cell-type-specific markers. We found that several modules, including M1, M3, M4, M7, M18, M19, M24, M25, M34, and M35, showed opposite correlation trends with AD and CAA pathology (Fig. 3A). Modules M1 and M3 were significantly enriched with astrocyte markers, M2 and M5 with neuron markers, and M15, M20, and M21 with endothelial cell markers. Gene Ontology (GO) enrichment analysis revealed that M1 and M3 were associated with glial cell-related functions, such as gliogenesis and glial cell differentiation, while M15, M20, and M21 were enriched in vasculogenesis and endothelial cell migration, and M2 and M5 were related to synapse organization (Fig. 3B).

To further investigate astrocyte responses in AD and CAA, we analyzed eigengene expression in M1 and M3. Compared with NC, eigengene expression was significantly elevated in AD but showed a decreasing trend in CAA (Fig. 3C), suggesting that astrocytes may promote AD progression by damaging neuronal synapses and contribute to CAA through effects on endothelial cell functions.

We next assessed the correlation between eigengene expression in M1 and M3 with levels of AD or CAA neuropathology. The “ABC” scoring system assigns descriptors (Not, Low, Intermediate, or High) to AD neuropathologic change [27]. We found a significant positive correlation between the eigengene expression in M1 (r = 0.544, p = 0.001) and M3 (r = 0.672, p = 0.009) with AD pathology (Fig. 3D). According to the Boston diagnostic criteria, CAA is defined by autopsy findings of lobar, cortical, or subcortical hemorrhages alongside severe vascular Aβ deposition, excluding other diagnoses [28]. In contrast to AD, eigengene expression in M1 (r = -0.462, p = 0.006) and M3 (r = -0.364, p = 0.034) was negatively correlated with CAA pathology (Fig. 3D), indicating distinct roles of astrocytes in AD and CAA neuropathological changes.

Gene expression alterations in astrocytes are critical in AD and CAA pathology [29,30]. Modules M1 and M3 shared 65 genes with DEGs in the CA4 subfield (Fig. 3E). Compared with NC, these 65 genes showed an upregulated trend in AD and a downregulated trend in CAA (Supplementary Fig. 3C). Protein-protein interaction (PPI) analysis showed strong interactions among these genes (Supplementary Fig. 3D). To identify crucial genes linked to distinct pathological changes, we used the maximal clique centrality (MCC) algorithm to highlight the top 10 DEGs with the highest scores (Fig. 3F). GLI2 (hub1), SMO (hub2), and CSF1 (hub3) were the top three, suggesting their expression patterns play key regulatory roles in promoting AD or CAA pathology (Fig. 3F and G)

**Figure 4.**
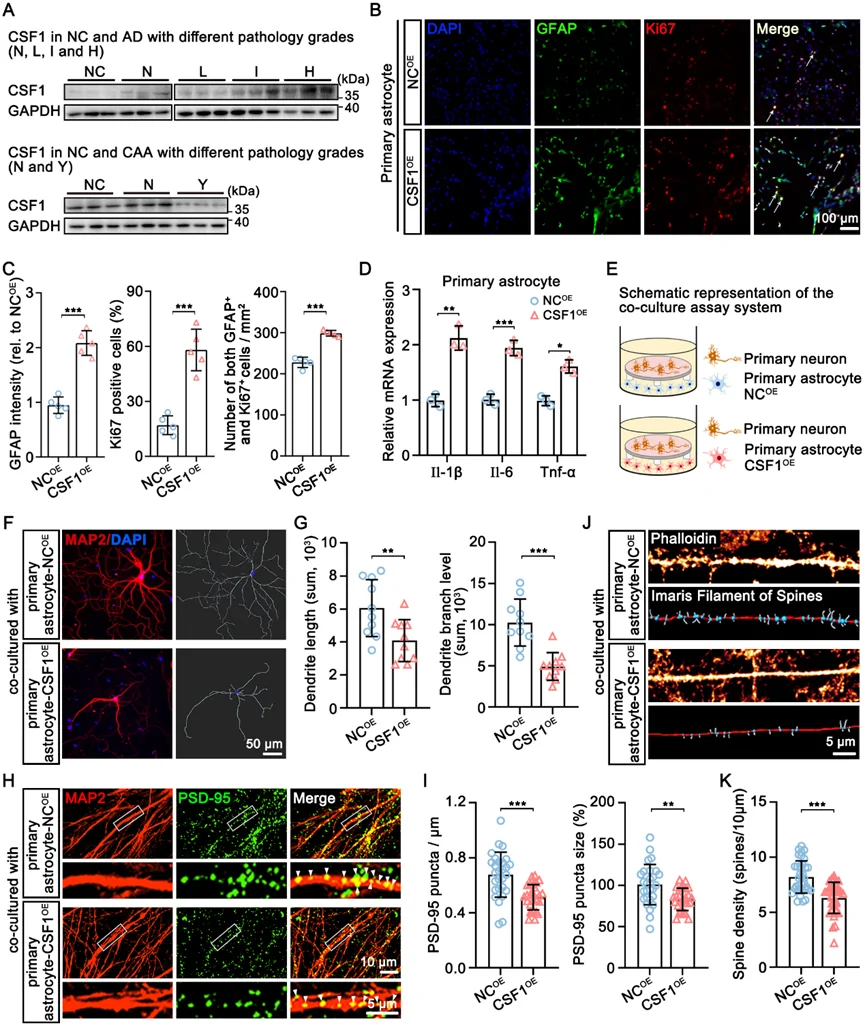
**Upregulation of CSF1 in astrocytes is neurotoxic**. (**A**) Detection of CSF1 protein in human brain tissue samples from NC (n = 3), AD (N: n = 3, L: n = 3, I: n = 3, H: n = 3) and CAA (N: n = 3, Y: n = 3) donors with different pathological grades. (**B-C**) Representative fluorescence micrographs and quantification of GFAP and Ki67 in primary astrocyte-NC^OE^ and primary astrocyte-CSF1^OE^ cells (n = 5). (**D**) Relative mRNA levels of *Il-1β* (n = 3), *Il*-6 (n = 3), and *Tnf-α* (n = 3) using qRT-PCR in primary astrocyte-NC^OE^ and primary astrocyte-CSF1^OE^. (**E**) Schematic diagram of co-culture system of primary hippocampal neurons with primary astrocytes. (**F**) Representative confocal images depict dendrite staining for primary rat hippocampal neurons co-cultured with primary astrocyte-NC^OE^ and with primary astrocyte-CSF1^OE^. (**G**) The statistics of dendrite area and length (n = 10 fields/group) in Figure 4F. (**H-I**) Representative images depict synaptic staining for dendrite MAP2 (red) and postsynaptic marker PSD-95 (green) in primary hippocampal neurons co-cultured with primary astrocyte-NC^OE^ and with primary astrocyte-CSF1^OE^ (H). Histograms depict relative density and size level of synapse (I). N = 28 fields/group. (**J-K**) Deconvolved images depicting spine densities labeled with phalloidin in primary hippocampal neurons co-cultured with primary astrocyte-NC^OE^ and with primary astrocyte-CSF1^OE^. Corresponding 3D-rendered views of spines were shown below (J). Statistic data showed alterations of spine density (K). N = 35 fields/group. *p < 0.05, **p < 0.01, ***p < 0.001, as determined by Student’s *t*-test for comparing two groups and one-way ANOVA for comparing multiple groups.

### Astrocytic CSF1 upregulation induces neuronal toxicity

Elevated CSF1 protein promotes microglial proliferation and induces neuroinflammation, aggravating the progression of AD-like pathology [31,32]. However, relatively few studies have focused on the potential role of astrocytic CSF1 in the distinct pathological and clinical manifestations of AD and CAA. We measured the protein and mRNA expression levels of CSF1 in brain tissues from NC, AD, and CAA donors. Compared with NC, CSF1 protein and mRNA levels were significantly increased in AD and markedly decreased in CAA (Fig. 4A, Supplementary Fig. 4A). Furthermore, CSF1 expression was positively correlated with the degree of AD pathology (r = 0.82, p = 0.0002), while it was negatively correlated with the degree of CAA pathology (r = -0.74, p = 0.02) (Supplementary Fig. 4B). These results were consistent with our transcriptomics data.

CSF1 protein was overexpressed in primary astrocytes and C8-D1A cells (mouse brain astrocyte) using a lentiviral transduction system. The qRT-PCR results showed a significant upregulation of *Csf1* expression (Supplementary Fig. 4C and D). Considering that the function of astrocytes involves gliogenesis (Fig. 3B), we evaluated whether CSF1 existed an effect on proliferation and activation of astrocytes. Glial fibrillary acidic protein (GFAP) and Ki67 were considered to be classic signatures of astrocyte activation and proliferation, respectively [20,33]. Overexpressed CSF1 led to an increased GFAP intensity and Ki67^+^ cells (Fig. 4B and C, Supplementary Fig. 4E). The sustained release of inflammatory cytokines caused by overactivated and proliferative astrocytes may precede the pathogenic cerebral changes relevant to AD [34]. The effect of elevated CSF1 on inflammatory responses was assessed. Our qRT-PCR demonstrated that *Csf1* significantly upregulated the expression of proinflammatory cytokines *Il* (interleukin) -*1β*, *Il-6* and tumor necrosis factor (*Tnf*) -*α* in primary astrocyte (Fig. 4D) and C8-D1A cells (Supplementary Fig. 4F).

Activation of astrocytes caused secretion of inflammatory factors, thereby reducing synaptic and neuronal health in AD [35,36]. Altered neuronal morphology accompanies functional damages during disease. Thus, we assessed morphological changes in rat hippocampal neurons cocultured with astrocyte-NC^OE^ or -CSF1^OE^ first (Fig. 4E), and MAP2 signals were used to parallel with neuronal morphology (Fig. 4F). We found that the dendrite area and length were significantly reduced under CSF1 overexpression (Fig. 4F and G). In addition, we detected changes in functional synapses (hub of neuronal networks) and their functional contacts dendritic spines *in vitro*. Postsynaptic density protein 95 (PSD-95) and phalloidin staining characterized postsynaptic membrane and dendritic spines, respectively. Neurons co-cultured with primary astrocytes-CSF1^OE^ had fewer and smaller PSD-95^+^ synapses than those cultured with astrocytes-NC^OE^ (Fig. 4H and I). Using Imaris and Image J, we performed deconvolution and three-dimensional (3D) reconstruction for captured dendrite spines images of neurons co-cultured with astrocytes-CSF1^OE^ and astrocytes-NC^OE^. Astrocytic CSF1 significantly decreased dendrite spines density. The density of dendrite spines was significantly reduced in neurons co-cultured with astrocyte-CSF1^OE^ compared with astrocyte-NC^OE^ (Fig. 4J and K). These data suggested that elevated CSF1 in astrocytes induced neuroinflammatory responses and damaged neuronal network communication.

### Astrocytic CSF1 deficiency damages vascular endothelial cell

**Figure 5.**
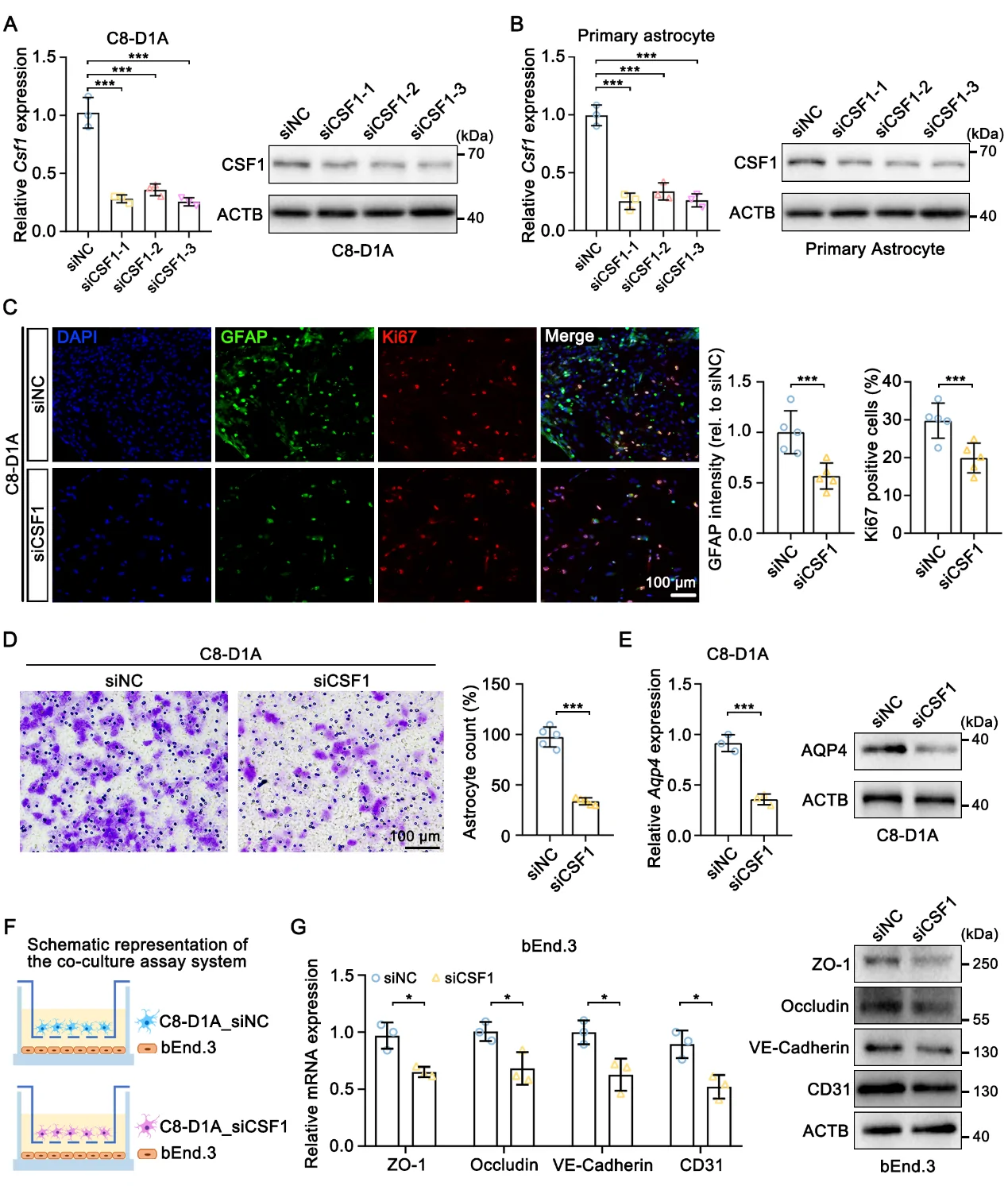
**Deficiency of CSF1 in astrocytes induces vascular endothelial cells dysfunction**. (**A**) Detection of CSF1 mRNA and protein levels using qRT-PCR and western blot, respectively, in C8-D1A-siNC cells (n = 3) or C8-D1A-siCSF1 cells (n = 3). (**B**) Detection of CSF1 transcription and protein levels using qRT-PCR and western blot, respectively, in primary rat astrocytes-siNC (n = 3) or primary rat astrocytes-siCSF1 (n = 3). (**C**) Immunofluorescence images and quantification of GFAP and Ki67 in C8-D1A-siNC (n = 5) and C8-D1A-siCSF1 cells (n = 5). (**D**) Representative images showing the effects of *Csf1* knockdown on C8-D1A cell migration. To quantify C8-D1A cell migration, crystal violet staining was conducted followed by absorbance at 570 nm measurement (n = 5). (**E**) Detection of AQP4 mRNA and protein levels in C8-D1A-siNC and C8-D1A-siCSF1 cells. (**F**) Schematic diagram of co-culture system of C8-D1A with bEnd.3 cells. (**G**) Detection of transcription and protein levels of endothelial cell marker CD31 and endothelial cell tight junction markers ZO-1, Occludin and VE-Cadherin in C8-D1A-siNC and C8-D1A-siCSF1 cells (n = 3). *p < 0.05, ***p < 0.001, as determined by Student’s *t*-test for comparing two groups and one-way ANOVA for comparing multiple groups.

Unlike AD, CSF1 expression in astrocytes of pathological CAA brain was markedly decreased. Based on our findings that astrocytes significantly correlated with vascular functions (Fig. 3B), we examined whether astrocytic CSF1 deficiency could support physiological endothelial barrier and defense properties. We first used small interfering RNA (siRNA) to construct non-targeting negative control (siNC) and CSF1 knockdown (siCSF1) C8-D1A cells and primary rat astrocytes. The qRT-PCR results showed that the intervention of siCSF1 reduced transcription level of *Csf1* by about 60-70% in both C8-D1A cells and primary astrocytes (Fig. 5A and B). Correspondingly, the CSF1 protein level was also detected to be significantly decreased in both cells (Fig. 5A and B).

To verify the effect of CSF1 protein reduction on astrocyte activation and proliferation, we stained GFAP and Ki67 in C8-D1A cells and primary astrocytes. Compared with the siNC group, CSF1 knockdown significantly reduced GFAP intensity and the number of Ki67-positive C8-D1A cells (Fig. 5C). A similar reduction was observed with siCSF1 treatment in primary astrocytes (Supplementary Fig. 5A). This decrease in astrocytes was associated with a reduction in the protective endfeet surrounding blood vessels. Additionally, CSF1 knockdown led to a significant reduction in the migration ability of both C8-D1A cells (Fig. 5D) and primary astrocytes (Supplementary Fig. 5B).

In humans and mice, AQP4 is predominantly localized to astrocyte endfeet near cerebral vasculature, playing a role in the clearance of brain fluid and solutes in CAA. AQP4 was significantly decreased in the brains of patients with severe CAA [37]. Therefore, we examined the effect of CSF1 knockdown in astrocytes on AQP4 expression. Both transcription and protein levels of AQP4 in C8-D1A-siCSF1 cells were significantly lower than in C8-D1A-NC cells (Fig. 5E). Similarly, CSF1 knockdown in primary astrocytes resulted in a marked decrease in AQP4 transcription and protein levels (Supplementary Fig. 5C).

**Figure 6.**
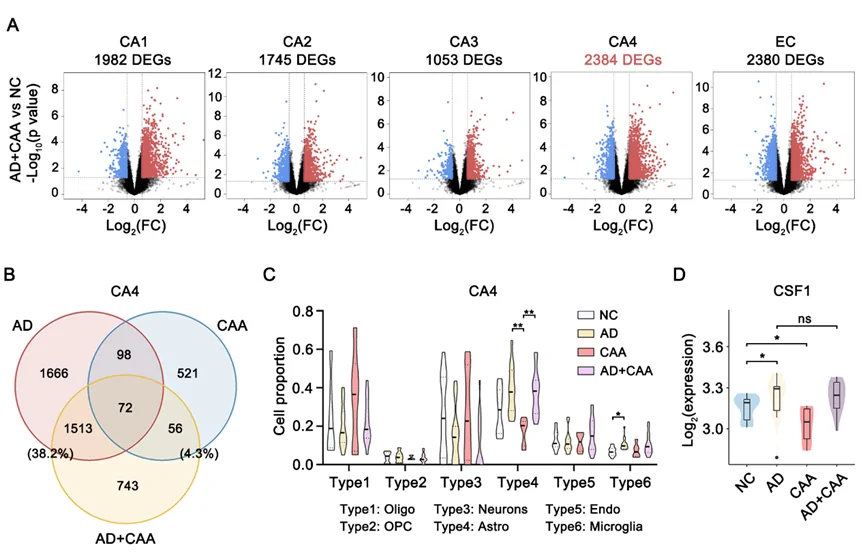
**Analysis and comparison of transcriptome data between AD+CAA and AD or CAA pathology**. (**A**) Volcano plots of DEGs for each subfield from individuals with AD+CAA pathology groups (n = 16); blue dots indicate downregulated genes and red dots indicate upregulated genes. (**B**) Venn diagram of DEGs across AD, CAA, and AD+CAA groups in CA4 subfield; and the proportion of DEGs shared by AD with AD+CAA, as well as CAA with AD+CAA groups. Colors represent different pathological groups. (**C**) Individual cell-type population proportions for NC, AD, CAA and AD+CAA groups in CA4 subfield. (**D**) Comparison of CSF1 expression in transcriptomic data of NC, AD, CAA and AD+CAA groups. *p < 0.05, **p < 0.01, ns = not significant, as determined by one-way ANOVA for comparing multiple groups.

In brain capillaries, endothelial cells are interconnected by transmembrane tight junction proteins that form the blood-brain barrier. Tight junction proteins create the paracellular barrier essential to the blood-brain barrier, and their loss has been associated with CAA severity [38]. Using C8-D1A and bEnd.3 (mouse brain microvascular endothelial cells), we established a co-culture to assess how the significant reduction of CSF1 in astrocytes may affect endothelial cell function (Fig. 5F). Transcription and protein levels of endothelial tight junction markers, including ZO-1, Occludin, VE-Cadherin, and the endothelial cell marker CD31, were significantly downregulated in bEnd.3 cells co-cultured with C8-D1A-siCSF1 cells (Fig. 5G). These findings suggest that the marked decrease in CSF1 in astrocytes compromises the endothelial cells' physiological barrier functions, potentially making blood vessels more susceptible to Aβ-induced damage and subsequent hemorrhage.

### Gene profile changes in AD+CAA pathology are more similar to those in AD

The AD and CAA pathways seem to diverge with respect to how they cause tissue injury: AD pathology promotes neuronal and synaptic loss via undefined mechanisms, whereas CAA generates focal tissue lesions via hemorrhagic and ischemic vascular brain injury [4]. However, a high proportion of individuals with AD also have comorbid CAA pathology. We also performed RNA-Seq analysis on CA1-CA4 and EC tissues from 16 donors with AD+CAA pathology (Supplementary Table S1). According to the screening criteria in Figure 1, we obtained 1,982 DEGs in CA1 subfield, 1,745 in CA2, 1,053 in CA3, 2,384 in CA4, and 2,380 in the EC. CA4 was the subfield with the largest number of DEGs (Fig. 6A). We also compared the differences in DEGs between the AD+CAA and AD, as well as between AD+CAA and CAA groups in the CA1-CA4 and EC regions. The proportion of shared DEGs between the AD and AD+CAA in the CA1 subfield was 28.7%, and that between the CAA and AD+CAA was 3.0%; in CA2, 22.9% with AD and 3.7% with CAA; in CA3, 16.1% with AD and 3.2% with CAA; in CA4, 38.2% with AD and 4.3% with CAA; in EC, 31.4% with AD and 2.9% with CAA (Fig. 6B, Supplementary Fig. 6A). This proportion was still the highest in the CA4 subfield, further verifying the importance of CA4 subfield in both AD and CAA pathologies.

Notably, among hippocampal-entorhinal five subfields, the proportion of shared DEGs between AD+CAA and AD was much higher than that between AD+CAA and CAA (Fig. 6B, Supplementary Fig. 6A). In addition, by referring to the single cell data in Figure 2, we tried to understand the changes in the proportion of CNS cell-types in the AD+CAA group. We found that there was no significant difference in the proportion of astrocytes in AD+CAA group and the AD group across all five subfields (Fig. 6C, Supplementary Fig. 6B, C, D and E). The alteration trend of CSF1 involved in regulating different pathologies of AD and CAA in AD+CAA was also the same as in AD (Fig. 6D). Taken together, these results suggested that the gene expression profile and changes in astrocytes in patients with AD combined with CAA pathology seem to be more similar with AD than with CAA.

## DISCUSSION

Complex interactions exist between CAA and AD pathophysiology, with shared mechanisms involving Aβ production, metabolism, and convective clearance from interstitial fluid via perivascular and intramural pathways [4]. In this study, we analyzed transcriptomes from hippocampal-entorhinal system subfields of postmortem human brain samples from individuals with AD, CAA, AD+CAA, and matched NC. Our aim was to comprehensively assess the pathological characteristics of AD and CAA using tissues from these regions.

Our transcriptome analysis revealed significant enrichment of AD- and CAA-related signatures and functions in the five hippocampal-entorhinal subfields, indicating distinct pathological changes across subfields. Distinct gene expression patterns were observed in response to AD and CAA, highlighting differential vulnerability within these regions. Notably, in CAA, the CA4 subfield exhibited the highest number of DEGs, followed by CA1, whereas in AD, CA4 showed the fewest DEGs, with CA1 exhibiting the most. This suggests that CA4 may be a critical intersection for AD and CAA pathogenic processes. CAA severity was associated with microinfarcts in CA1 [39], while pyramidal neurons in CA1, known for their intrinsic excitability and synaptic plasticity, are highly vulnerable to AD [40]. However, CA4 has been relatively understudied in postmortem AD pathology [41], and few studies focus on CAA in this region. Our findings underscore the important involvement of CA4 in both AD and CAA.

Neurodegeneration may alter cellular composition [42], and single-cell RNA sequencing offers insights into cellular heterogeneity by profiling thousands of individual cells [43]. A comparative analysis of brain cell types in AD and CAA may improve our understanding of these diseases. Our results indicated a significant increase in astrocytes in CA4 of individuals with AD and a significant decrease in those with CAA, suggesting a regulatory role for astrocyte numbers in the progression of each pathology. Under pathological conditions, astrocytes undergo morphological and functional changes collectively referred to as reactive astrocytes [44]. Reactive astrocytes are common in postmortem AD brain tissues in areas with high Aβ or tau pathology [45,46], while in CAA, astrocytes interact closely with blood vessels, as they are integral to the neurovascular unit (NVU) [47,48]. Our data indicated that astrocytes were positively correlated with AD neuropathology and negatively correlated with CAA pathology, suggesting that astrocytes may mediate responses to these distinct pathologies.

Weighted Gene Co-expression Network Analysis (WGCNA), a systems biology approach, groups co-expressed genes potentially involved in similar biological processes and has proven effective in identifying hub genes for neurological disorders, including AD and CAA [49,50]. In this study, we combined WGCNA with single-cell data to analyze transcriptomes from the hippocampal-entorhinal system in AD and CAA. Differential expressions of cell type-related eigengenes suggested distinct disease associations for specific cell types [51]. Genes from AD and CAA samples were enriched in astrocyte-, endothelial cell-, and neuron-specific markers. Notably, astrocyte-enriched genes showed opposite expression trends in AD and CAA.

Three genes, GLI2, SMO, and CSF1, showed differential expression between AD or CAA and NC groups. GLI2, a zinc finger protein, mediates Hedgehog (Hh) signaling, regulating genes involved in cell proliferation and differentiation [52]. Although GLI2 is a known neural development regulator [53,54], its role in AD and CAA is largely unexplored. SMO, a key G protein-coupled receptor (GPCR) in the Sonic Hedgehog (SHH) pathway, promotes neuroprotection and recovery in neurological diseases [56]. Studies have shown that SMO upregulation in AD-related regions mitigates neuroinflammation and Aβ-induced memory loss [57], but its relationship with CAA remains unexplored. This led us to analyze CSF1’s distinct roles in AD and CAA. CSF1, a growth factor expressed by neurons and astrocytes, maintains synaptic, neurotrophic, and microglial homeostasis at baseline levels [58]. Our results showed that CSF1 protein levels increased with AD pathology severity, whereas they were significantly reduced in CAA. CSF1 expression correlated positively with AD pathology and negatively with CAA. Integrating data from fields like neuroimaging and cognitive neuroscience could further illuminate how CSF1 influences brain structure and function in AD and CAA.

Previous studies have shown that CSF1 expression is upregulated in AD and AD-like transgenic mice, playing an important role in microglial proliferation caused by pathological activation [59]. Microglial elimination by inhibition of CSF1/CSF1R signaling in AD disease models ameliorated neurodegeneration and functional recovery [60]. In a report at ALZFORUM in 2022, researchers proposed that knockout of CSF1 receptor CSF1R did not affect Aβ plaque formation in AD mice, but caused a significantly increased plaques surrounding endothelial cells. Further analysis showed that decreased PDGF-β and TGF-β induced by microglial deficiency led to extensive blood vessel damage (www.alzforum.org/news/research-news/sans-microglia-mice-develop-caa-and-die-young). However, astrocytic CSF1 alterations in addressing AD and CAA pathologies were unclear. Here, we show that astrocytic CSF1 upregulation in AD correlates with an inflammatory phenotype, which induces damage to neurons. CSF1 deficiency in astrocytes causes decreased expression of the brain water-channel protein AQP4, weakened vascular protection, and significantly reduced expression of tight junction proteins in endothelial cells. These findings imply that decreased CSF1 expression in CAA may lead to vascular dysfunction and blood-brain barrier disruption, promoting amyloid deposition within cerebral vessels. These contrasting roles of CSF1 highlight its potential as a modulatory factor in the neurovascular unit, influencing both neuroinflammatory responses in AD and vascular integrity in CAA. The upregulation of CSF1 in AD could be seen as a detrimental response, amplifying local inflammation and contributing to neuronal damage. In contrast, downregulation of CSF1 in CAA might reflect an insufficient astrocytic support to cerebral vessels, leading to vascular pathology and possibly exacerbating amyloid accumulation.

In conclusion, we conducted a robust transcriptomic study of the human hippocampal-entorhinal system subfields in AD and CAA. By evaluating differentially expressed genes across various subfields and disease states, and integrating single-cell data, we found that changes in astrocyte proportions in the hippocampal CA4 subfield may be crucial to blood vessel or neuron damage. Additionally, our findings suggest that CSF1-mediated astrocyte functional changes are involved in both AD and CAA pathologies. Future studies should explore the therapeutic potential of modulating CSF1 levels to mitigate its harmful effects in AD while preserving its essential role in vascular health. Understanding the specific pathways through which CSF1 influences astrocyte behavior and interacts with other cells in the neurovascular unit could yield valuable insights for targeted treatments in AD and CAA. This approach may uncover novel strategies to alleviate the distinct yet overlapping pathologies of these complex neurodegenerative conditions.

## Supplementary Materials

The Supplementary data can be found online at: https://www.aginganddisease.org/EN/10.14336/AD.2024.10530.
