# Supplementary material for: Differential Roles of Astrocytic CSF1 in Alzheimer's Disease and Cerebral Amyloid Angiopathy: Insights from Transcriptomic Analysis
Source: Aging Dis. 2024 Nov 5;16(5):3137–53. doi: 10.14336/AD.2024.10530 (PMC12339160; doi:10.14336/AD.2024.10530)
Supplement: Supplementary file 1 [file AD-16-5-3137-s.pdf]

SUPPLEMENTARY DATA

Differential Roles of Astrocytic CSF1 in Alzheimer's Disease and Cerebral Amyloid Angiopathy: Insights from Transcriptomic Analysis

Chunyuan Li, Yashuang Chen, Shiqi Luo, Yan Yang, Xinnan Liu, Sijie Li, Wei Ge, Cong Han

© 2024. Li C et al. Published online at http://www.aginganddisease.org/EN/10.14336/AD.2024.10530

SUPPLEMENTARY DATA

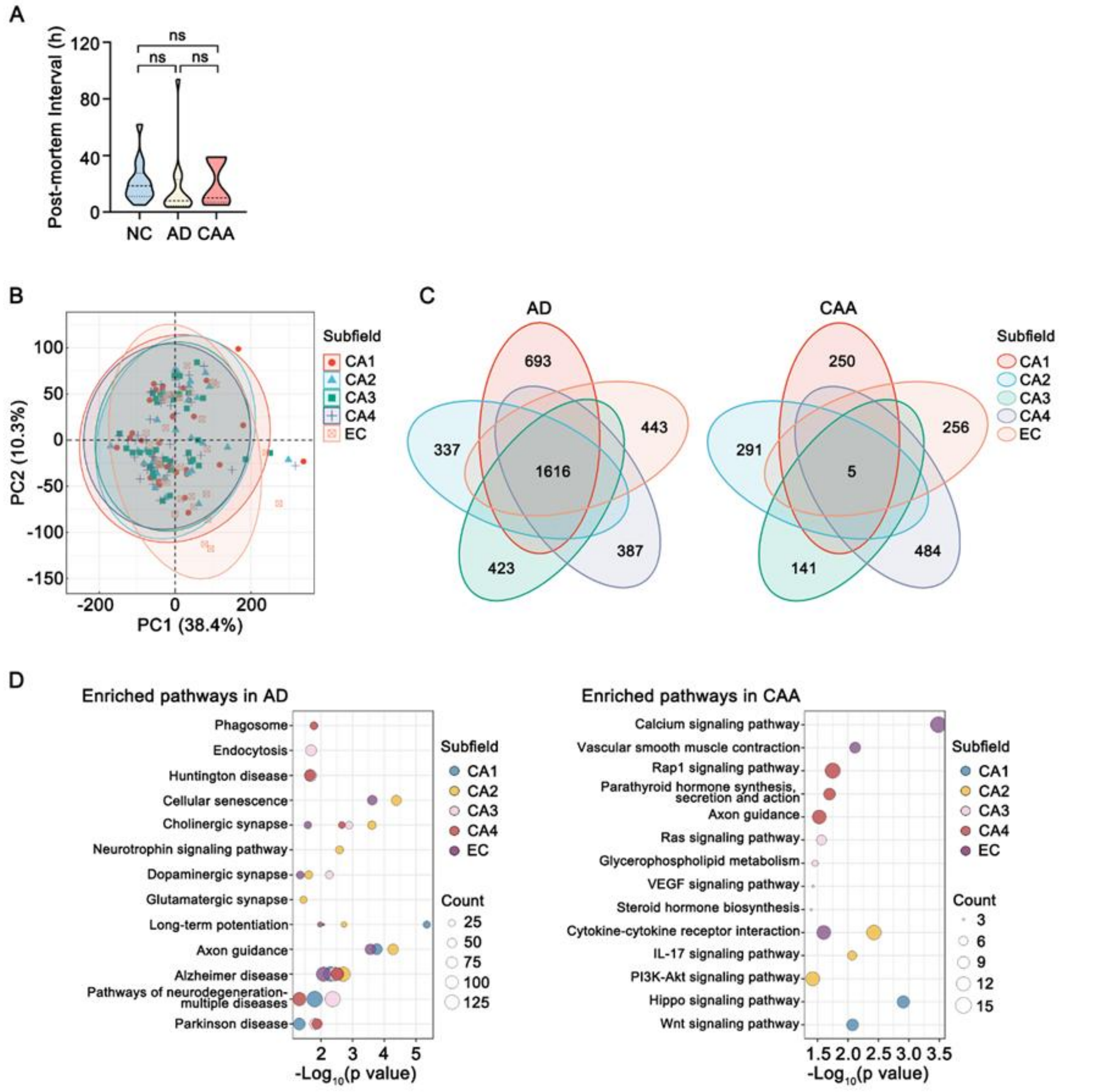

Supplementary Figure 1. Transcriptomic analyses of hippocampal–entorhinal system subfields from individuals with AD or CAA pathology. (A) Statistical analysis of PMI of brain donors among NC (n = 13), AD (n = 15) and CAA (n = 6) groups. **(B)** PCA plot of hippocampal–entorhinal system subfields RNA-Seq data. Each color indicated a subfield and each dot indicated a sample. **(C)** Venn diagrams of DEGs in hippocampal–entorhinal system subfields of AD (left) and CAA (right) groups. **(D)** KEGG enrichment analyses of DEGs in hippocampal–entorhinal system subfields of AD (left) and CAA (right) groups.

© 2024. Li C et al. Published online at http://www.aginganddisease.org/EN/10.14336/AD.2024.10530

SUPPLEMENTARY DATA

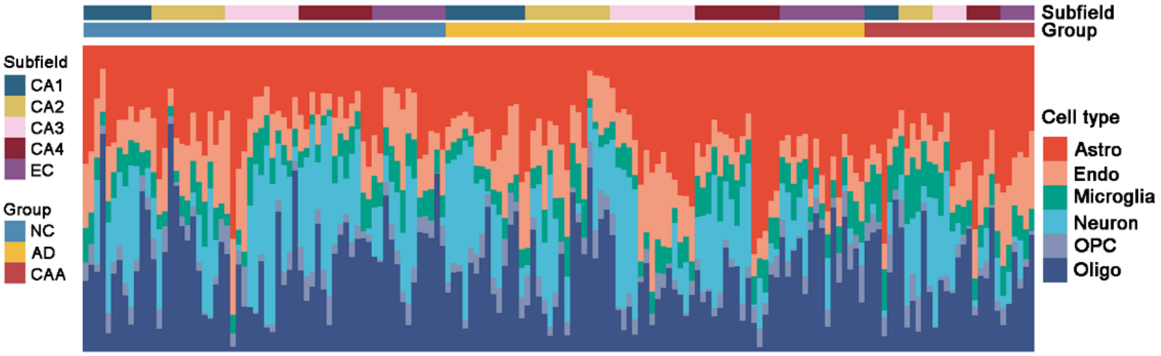

Supplementary Figure 2. The fractions of six cell types (Oligo, OPC, Neurons, Astro, Endo and Microglia) in each sample. Oligo = Oligodendrocyte, OPC = Oligodendrocyte precursor Cell, Astro = Astrocyte, Endo = Endothelial cell.

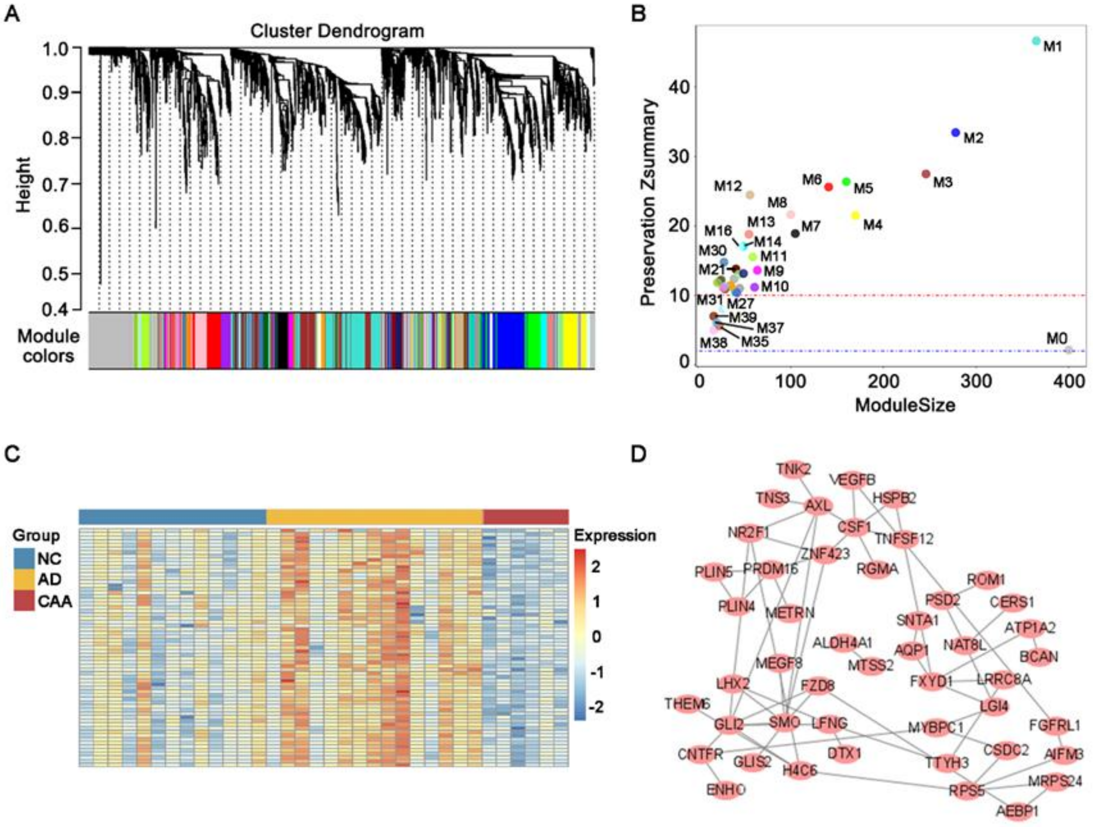

Supplementary Figure 3. WGCNA across NC, AD and CAA groups and screening of astrocyte-related genes. **(A)** WGCNA results and clustering dendrograms. **(B)** Illustration of modules preservation statistics. **(C)** Hierarchical cluster analysis of gene expression profiles across NC, AD and CAA samples. **(D)** PPI analysis of overlapping DEGs in Figure 3E.

© 2024. Li C et al. Published online at http://www.aginganddisease.org/EN/10.14336/AD.2024.10530

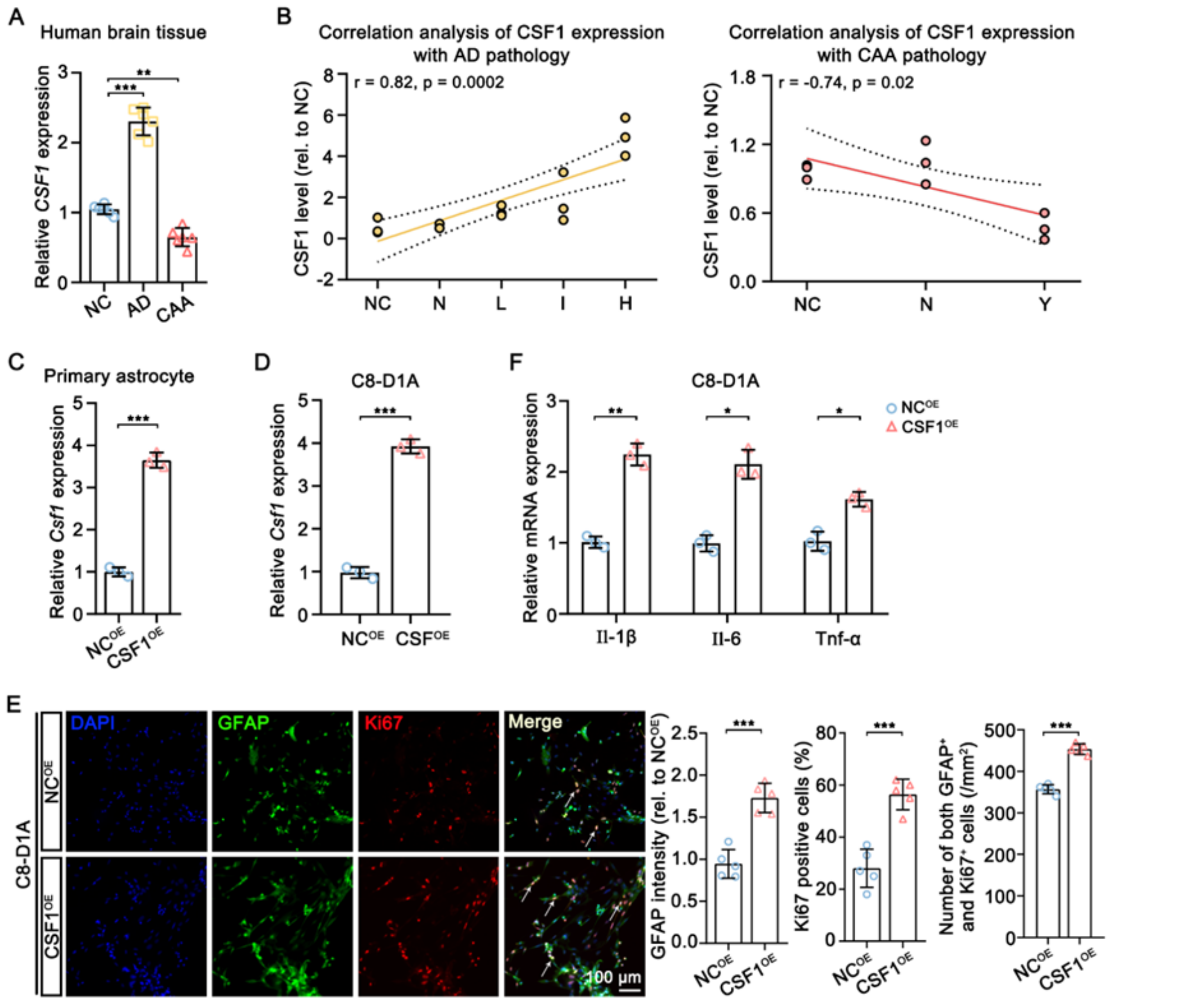

Supplementary Figure 4. Changes of inflammatory factors and proliferation ability in C8-D1A overexpressing CSF1. **(A)** Validation of CSF1 expression in human hippocampal CA4 subfield tissues with no- (NC, n = 6), AD- (n = 6) and CAA- (n = 6) pathology using qRT-PCR. **(B)** Correlation analyses of CSF1 expression with AD and CAA pathology. **(C)** Validation of overexpression efficiency of *Csf1* in primary astrocytes using qRT-PCR (n = 3). **(D)** Validation of overexpression efficiency of *Csf1* in C8-D1A cells using qRT-PCR (n = 3). **(E)** Representative fluorescence micrographs and quantification of GFAP and Ki67 in C8-D1A-NC^{OE} and C8-D1A-CSF1^{OE} cells (n = 5). **(F)** Relative mRNA levels of *Il-1β*, *Il*-6, and *Tnf-α* using qRT-PCR in C8-D1A-NC^{OE} and C8-D1A-CSF1^{OE} cells (n = 3). *p < 0.05, **p < 0.01, ***p < 0.001, as determined by Student's *t*-test for comparing two groups.

© 2024. Li C et al. Published online at http://www.aginganddisease.org/EN/10.14336/AD.2024.10530

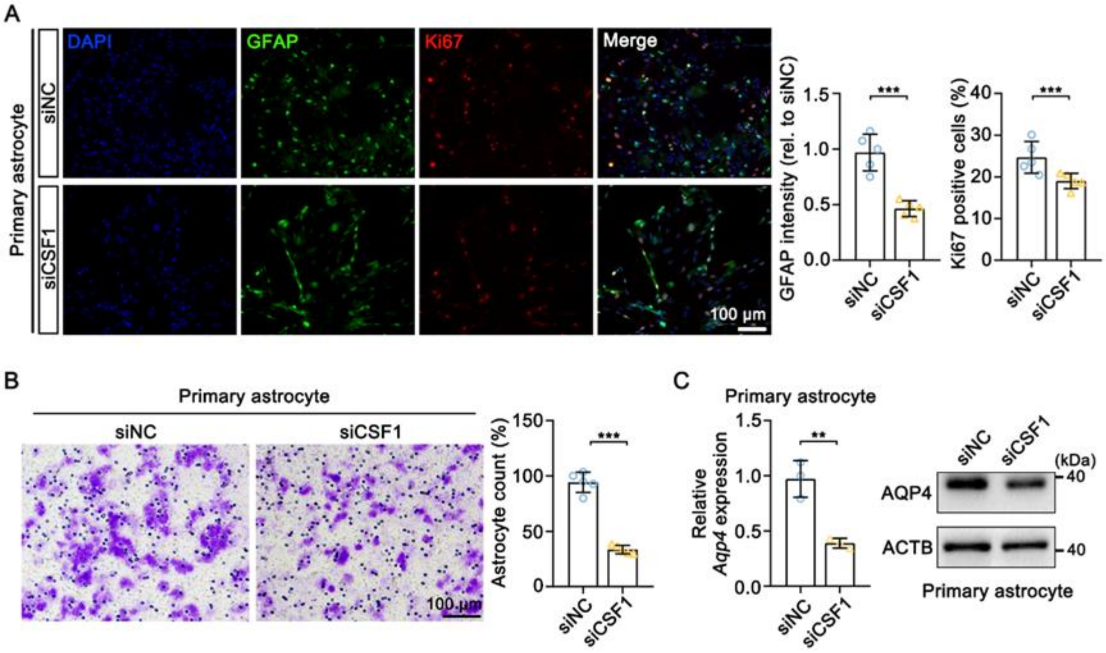

Supplementary Figure 5. The impact of CSF1 reduction in primary astrocytes on endothelial cells. (A) Immunofluorescence images and quantification of GFAP and Ki67 of primary rat astrocyte-siNC and primary astrocyte-siCSF1 cells (n = 5). **(B)** Representative images showing the effects of *Csf1* knockdown on primary rat astrocytes migration. To quantify primary astrocytes migration, crystal violet staining was performed followed by absorbance measurement at 570 nm (n = 5). **(C)** Detection of AQP4 mRNA and protein levels in primary astrocyte -siNC and primary astrocyte-siCSF1 cells (n = 3).

© 2024. Li C et al. Published online at http://www.aginganddisease.org/EN/10.14336/AD.2024.10530

SUPPLEMENTARY DATA

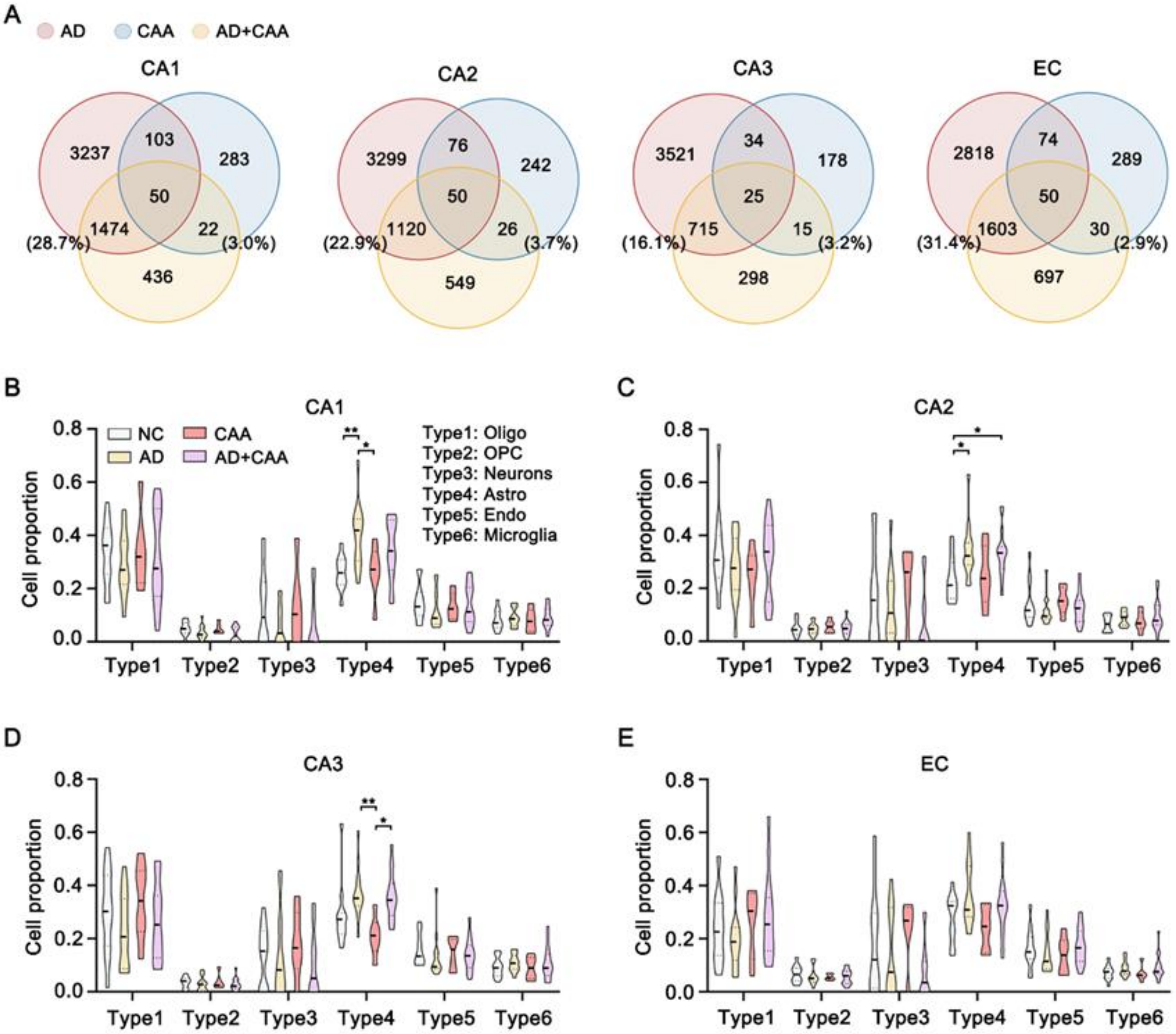

Supplementary Figure 6. Analysis and comparison of transcriptome data between AD+CAA and AD, as well as between AD+CAA and CAA. (A) Venn diagrams of DEGs across AD, CAA, and AD+CAA groups in CA1, CA2, CA3, and EC; and the proportion of DEGs shared by AD or CAA and AD+CAA groups. **(B-E)** Individual cell-type population proportions for NC, AD, CAA, and AD+CAA groups in CA1, CA2, CA3 and EC subfield. *p < 0.05, **p < 0.01, as determined by one-way ANOVA for comparing multiple groups.

© 2024. Li C et al. Published online at http://www.aginganddisease.org/EN/10.14336/AD.2024.10530

SUPPLEMENTARY DATA

Supplementary Table 1. Basic information and associated traits of human brain donors.

Sample ID	Sex	Age	ABC score	CAA score	Group	PMI (h)
01	M	86	L	N	NC	37.5
02	F	86	N	N	NC	14
03	F	96	N	N	NC	32
04	F	86	N	N	NC	23
05	M	75	L	N	NC	62
06	M	62	N	N	NC	18.5
07	F	62	N	N	NC	10
08	M	64	N	N	NC	12
09	F	89	N	N	NC	22.5
10	M	86	L	N	NC	20.5
11	M	86	N	N	NC	5
12	M	86	N	N	NC	14
13	M	71	N	N	NC	10
14	M	74	I	N	AD	28
15	F	81	H	N	AD	8
16	F	90	H	N	AD	93.8
17	F	89	I	N	AD	12.3
18	F	88	I	N	AD	7
19	F	89	I	N	AD	6.5
20	M	77	I	N	AD	4
21	M	89	I	N	AD	11
22	F	75	I	N	AD	8
23	M	94	I	N	AD	4.3
24	F	93	I	N	AD	7.3
25	F	86	I	N	AD	4
26	M	85	H	N	AD	23
27	F	90	H	N	AD	3.5
28	M	85	H	N	AD	26
29	M	83	N	Y	CAA	38
30	M	86	L	Y	CAA	10
31	M	95	N	Y	CAA	5
32	F	74	N	Y	CAA	4
33	F	96	L	Y	CAA	9
34	M	72	N	Y	CAA	39
35	F	91	H	Y	AD+CAA	3
36	F	86	I	Y	AD+CAA	8
37	F	84	I	Y	AD+CAA	4
38	F	86	I	Y	AD+CAA	18
39	M	102	I	Y	AD+CAA	46
40	F	91	I	Y	AD+CAA	12
41	M	91	I	Y	AD+CAA	16.5
42	F	86	I	Y	AD+CAA	5.5
43	M	86	I	Y	AD+CAA	7
44	M	98	H	Y	AD+CAA	3
45	M	88	H	Y	AD+CAA	3.3
46	F	88	I	Y	AD+CAA	4.5
47	F	85	H	Y	AD+CAA	4.5
48	M	89	H	Y	AD+CAA	4.5
49	M	89	H	Y	AD+CAA	3
50	M	89	H	Y	AD+CAA	4.5

PMI: Post-mortem Interval

© 2024. Li C et al. Published online at http://www.aginganddisease.org/EN/10.14336/AD.2024.10530

SUPPLEMENTARY DATA

Supplementary Table 2. Statistical analysis for experimental data.

Figures	Test object	Statistical analysis	Values of F	Degree of freedom	Exact values of p
**Fig. 2F**	GFAP⁺ cell	one-way ANOVA with Turkey post hoc test for multiple groups	381.5	DFn=2, DFd=6	< 0.0001, 0.0047
**Fig. 4A**	CSF1	one-way ANOVA with Turkey post hoc test for multiple groups	219.5	DFn=2, DFd=15	< 0.0001, 0.0004
**Fig. 4B**	CSF1	Two-tailed unpaired Student's t-test	2.844	DFn=2, DFd=2	< 0.0001
**Fig. 4D**	GFAP	Two-tailed unpaired Student's t-test	2.228	DFn=4, DFd=4	< 0.0001
	Ki67	Two-tailed unpaired Student's t-test	17.45	DFn=5, DFd=5	< 0.0001
	Both GFAP and Ki67	Two-tailed unpaired Student's t-test	3.211	DFn=4, DFd=4	< 0.0001
**Fig. 4E**	IL-1β	Two-tailed unpaired Student's t-test	3.812	DFn=2, DFd=2	0.0014
	IL-6	Two-tailed unpaired Student's t-test	2.028	DFn=2, DFd=2	0.0007
	TNF-α	Two-tailed unpaired Student's t-test	1.837	DFn=2, DFd=2	0.002
**Fig. 4H**	Dendrite length	Two-tailed unpaired Student's t-test	1.841	DFn=9, DFd=9	0.0093
	Dendrite branch	Two-tailed unpaired Student's t-test	2.88	DFn=9, DFd=9	< 0.0001
**Fig. 4J**	PSD-95 puncta number	Two-tailed unpaired Student's t-test	3.177	DFn=27, DFd=27	< 0.0001
	PSD-95 puncta size	Two-tailed unpaired Student's t-test	3.268	DFn=27, DFd=27	0.0014
**Fig. 4L**	Spine density	Two-tailed unpaired Student's t-test	1.083	DFn=34, DFd=34	< 0.0001
**Fig. 5A**	CSF1	one-way ANOVA with Turkey post hoc test for multiple groups	71.12	DFn=3, DFd=8	< 0.0001, < 0.0001, < 0.0001
**Fig. 5B**	CSF1	one-way ANOVA with Turkey post hoc test for multiple groups	70.96	DFn=3, DFd=8	< 0.0001, < 0.0001, < 0.0001
**Fig. 5C**	GFAP	Two-tailed unpaired Student's t-test	2.721	DFn=4, DFd=4	0.0045
	Ki67	Two-tailed unpaired Student's t-test	1.387	DFn=4, DFd=4	0.0066
**Fig. 5D**	Astrocyte	Two-tailed unpaired Student's t-test	8.294	DFn=4, DFd=4	< 0.0001
**Fig. 5E**	AQP4	Two-tailed unpaired Student's t-test	2.42	DFn=2, DFd=2	0.0006
**Fig. 5G**	ZO-1	Two-tailed unpaired Student's t-test	6.494	DFn=2, DFd=2	0.011
	Occludin	Two-tailed unpaired Student's t-test	2.889	DFn=2, DFd=2	0.0274
	VE-Cadherin	Two-tailed unpaired Student's t-test	1.816	DFn=2, DFd=2	0.0212
	CD31	Two-tailed unpaired Student's t-test	1.372	DFn=2, DFd=2	0.0154
**Fig. S4A**	CSF1	Two-tailed unpaired Student's t-test	1.597	DFn=2, DFd=2	< 0.0001
**Fig. S4B**	GFAP	Two-tailed unpaired Student's t-test	1.054	DFn=4, DFd=4	< 0.0001
	Ki67	Two-tailed unpaired Student's t-test	1.574	DFn=4, DFd=4	0.0001
	Both GFAP and Ki67	Two-tailed unpaired Student's t-test	1.298	DFn=4, DFd=4	< 0.0001
**Fig. S4C**	IL-1β	Two-tailed unpaired Student's t-test	3.726	DFn=2, DFd=2	0.0003
	IL-6	Two-tailed unpaired Student's t-test	3.151	DFn=2, DFd=2	0.0012
	TNF-α	Two-tailed unpaired Student's t-test	1.675	DFn=2, DFd=2	0.0038
**Fig. S5A**	GFAP	Two-tailed unpaired Student's t-test	5.273	DFn=4, DFd=4	0.0002
	Ki67	Two-tailed unpaired Student's t-test	17.55	DFn=5, DFd=5	< 0.0001
**Fig. S5B**	Astrocyte	Two-tailed unpaired Student's t-test	5.739	DFn=4, DFd=4	< 0.0001
**Fig. S5C**	AQP4	Two-tailed unpaired Student's t-test	14.34	DFn=2, DFd=2	0.0023

© 2024. Li C et al. Published online at http://www.aginganddisease.org/EN/10.14336/AD.2024.10530
